# Timing of Surgery and Preoperative Predictors of Surgical Site Infections for Patients with Depressed Skull Fractures in a Sub-Saharan Tertiary Hospital: A Prospective Cohort Study

**DOI:** 10.1089/neur.2024.0088

**Published:** 2024-09-20

**Authors:** Hervé Monka Lekuya, Jelle Vandersteene, Larrey Kasereka Kamabu, Rose Nantambi, Ronald Mbiine, Anthony Kirabira, Fredrick Makumbi, Stephen Cose, David Patrick Kateete, Mark Kaddumukasa, Edward Baert, Moses Galukande, Jean-Pierre Okito Kalala

**Affiliations:** ^1^ Department of Surgery/Neurosurgery, College of Health Sciences, Makerere University, Kampala, Uganda.; ^2^ Department of Human Structure and Repair/Neurosurgery UZ Gent, Ghent University, Ghent, Belgium.; ^3^ School of Public Health, College of Health Sciences, Makerere University, Kampala, Uganda.; ^4^ Medical Research Council/London School of Hygiene & Tropical Medicine, MRC Uganda, Entebbe ,Uganda.; ^5^ Department of Molecular Biology, College of Health Sciences, Makerere University, Kampala, Uganda.; ^6^ Department of Internal Medicine, College of Health Sciences, Makerere University, Kampala, Uganda.

**Keywords:** depressed skull fractures, pneumocranium, surgical timing, surgical site infection

## Abstract

Surgical site infections (SSIs) remain a major cause of life-threatening morbidity following surgery for depressed skull fractures (DSFs) among patients with traumatic brain injury (TBI). The timing of the surgery for DSF has been questioned as a risk of SSI without a clear cutoff. We aimed to compare the risk of SSI within 3 months between surgery done before versus after 48 h of injury and with its preoperative predictors. We conducted a prospective cohort study at Mulago Hospital, Uganda. Patients with mild-to-moderate TBI with DSF were followed up perioperatively from the operating time up to 3 months. The outcome variables were the incidence risk of SSI, types of SSI, microbial culture patterns of wound isolates, and hospital length of stay. We enrolled 127 patients with DSF, median age = 24 (interquartile range [IQR] = 17–31 years), 88.2% (112/127) male, and assault victims = 53.5%. The frontal bone involved 59%, while 50.4% had a dural tear. The incidence of SSI was 18.9%, mainly superficial incisional infection; Gram-negative microorganisms were the most common isolates (64.7%). The group of surgical intervention >48 h had an increased incidence of SSI (57.3% vs. 42.7%, *p* = 0.006), a longer median of postoperative hospital stay (8[IQR = 6–12] days versus 5 [IQR = 4–9], [*p* < 0.001]), and a higher rate of reoperation (71.4% vs. 28.6%, *p* = 0.05) in comparison with the group of ≤48 h. In multivariate analysis between the group of SSI and no SSI, surgical timing >48 h (95% confidence interval [CI], 1.25–6.22), pneumocranium on computed tomography [CT] scan (95% CI: 1.50–5.36), and involvement of air sinus (95% CI: 1.55–5.47) were associated with a >2.5-fold increase in the rate of SSI. The SSI group had a longer median hospital stay (*p* value <0.001). The SSI risk in DSF is high following a surgical intervention >48 h of injury, with predictors such as the frontal location of DSF, pneumocranium on a CT scan, and involvement of the air sinus. We recommend early surgical intervention within 48 h of injury.

## Introduction

Depressed skull fracture (DSF) is a type of skull fracture in traumatic brain injury (TBI), in which a segment of the vault of the skull is depressed below its anatomical position, and its edges may become locked underneath the adjacent intact bone^1^; it results fom a high-energy rapid compression force applied to a small area of the skull.^1,2^ This type of fracture is often associated with intracranial injuries, which include vascular damage and direct brain damage^3–5^; it may constitute an abnormal area of direct physical irritation of the pulsating brain. DSF accounts for 6–16% of admitted patients with TBI and is often managed with the surgical strategy of debridement and elevation, with or without additional surgical corrections whenever applicable. They can be classified as simple (closed) or compound (open) when there is a skin laceration communicating the external environment with the cranial cavity.^6^ Even if a DSF is compound, a nonoperative approach to the bone breach could be a treatment option if there is no evidence of a violation of the dura mater, significant intracranial hematoma, depressed bone fragments of more than 1 cm below the inner table of the skull, frontal sinus involvement, gross cosmetic deformity, wound infection, or gross wound contamination.^6–9^ However, there is an increased risk of morbidity of surgical site infections (SSIs) following the surgical management up to 3 months with eventual life-threatening complications such as meningitis, meningeal empyema, cerebritis, and brain abscess.^5,6,10,11^ SSI often requires immediate medical/surgical reintervention, and death occurs if not treated quickly and energetically.^12^ Indeed, the occurrence of SSI from cranial neurosurgical procedures is multifactorial: postoperative bleeding, diabetes type 2, operations performed by nonexperienced surgeons, duration of surgery, recent operation, concurrent extracranial infections, respiratory failure, hemodynamic instability, low Glasgow Coma Scale (GCS), and others known to be contributing to the development of SSI following cranial surgery.^13–17^ However, the timing of surgery for DSF and the patterns of infection remain poorly studied among hemodynamically stable nonsevere patients. Many retrospective studies report no clear benefit of the 24-h timing of surgery of DSF, but one systematic review study done two decades ago advocated early surgery as the safest timing without a clear cutoff of the timing of intervention,^6^ and a recent one even did not find any significant difference in the risk of SSI between conservative versus surgery of compound DSF.^8^ Indeed, preoperative predictors of SSI of DSF surgery, for instance, the surgical timing and the clinical-radiological parameters, are yet to be studied and analyzed prospectively due to inclusion into previous studies of known confounders of SSI and poor outcomes such as very sick patients and comorbidities.^6,8,18^ Looking at the previously published guidelines or review studies for TBI management regarding DSF, there is no clear consensus or recommended cutoff timing of surgery for DSF regarding the risk of SSI due to a lack of clinical studies with a higher level of evidence.^6,19,20^ Except for some management strategies that have been documented based on previous retrospective studies and clinical judgment with controversies on the recommended cutoff of the surgical timing, there are very few prospective studies done to ascertain the preoperative predictors of SSI in DSFs, yet those are the key informants for the prognosis of surgical management and also for possible parameters of updating clinical guidelines of TBI management, especially when it comes in the context of limited resource settings with a higher burden of patients with TBI, as it is the current case in sub-Saharan Africa (SSA). We aimed to compare the risk of developing SSI within 3 months between surgery of DSF done before and after 48 h of injury in patients with nonsevere TBI and the preoperative predictors of the occurrence of SSI.

## Methods

### Design, setting, and participants

This was a prospective cohort study conducted at Mulago National Referral Hospital (MNRH), Kampala, Uganda. The MNRH is the largest public tertiary hospital in Uganda and also the teaching hospital of Makerere University College of Health Sciences. Its Accident & Emergency (A&E) Department serves as an equivalent of a “level 1 trauma center facility” in Uganda and treats a high volume of trauma patients referred from all over the country.^21–23^ The study participants were patients with TBI of all ages with DSF evidenced on a brain computed tomography (CT) scan exclusively, admitted in the A&E Department or at the referring hospital within 6 h of injury, with a postresuscitation GCS above 8, with SpO_2_ > 94% in room air, hemodynamically stable, with a hemoglobin level above 9 g/dL, operated for an elevation of DSF, and whose informed written consent was obtained. We excluded patients with evidence of scalp infection, gross wound contamination with or without exposed bone fragments/fungi cerebri, multiple (more than two) scalp wounds or skin loss, with other signs of infections before surgery, with a penetrating mechanism, with a DSF over the midline on the posterior 2/3 of the superior sagittal sinus, patients readmitted after an attempt at nonsurgical management, and with a history of head trauma, brain surgery, steroids treatment, or with comorbidities. Also excluded are patients with large extra-axial hemorrhages, considered the primary surgical indication, and those operated on for emergency decompressive craniectomy as the surgical strategy.

### Sampling and study variables

Patients were recruited consecutively by convenience until the calculated sample size was attained with a relatively equal distribution of the two groups of interest (surgical intervention before vs. after 48 h of injury).

The main exposure variable was the timing of surgery, which was defined as early surgical intervention when done before 48 h after injury and delayed surgical intervention when done after 48 h following injury. The surgical timing cutoff of before 48 h (early surgery) versus after 48 h (delayed surgery) was chosen initially based on the cutoff of early versus delayed surgical intervention regarding the post-traumatic inflammatory process in polytrauma patients^24^ and posteriorly maintained based on the “no statistical significance” in this study subanalysis of surgical intervention between before and after 24 h of injury.

Other independent variables included the sociodemographic characteristics (age and sex), mechanisms of injury, association with long bone fracture, postresuscitation GCS score, neurological deficit, post-traumatic seizure (immediate and early), preoperative hemoglobin, type of DSF (simple or compound), location (frontal, parietal, temporal, or occipital) of DSF, head CT scan findings (underlying cerebral contusion, extra-axial hemorrhage, pneumocranium, involvement of the air sinus, status of the basal cisterns, and the presence of midline shift), American Society of Anesthesiologists (ASA) classification, duration of anesthesia, duration of surgery, and intraoperative dural tear.

The main dependent variables (primary outcome variables) were the occurrence of SSI within 3 months, which was measured as a dichotomous outcome (SSI vs. no SSI), types of SSI (superficial incisional infection, cranial osteomyelitis, epidural/subdural empyema, meningitis, or brain abscess), and culture-sensitivity patterns of wound, cerebrospinal fluid (CSF), or abscess isolates.

The secondary outcome variables were the postoperative hospital length of stay (in days), total hospital length of stay (in days), and overall hospital outcome at 3 months after the initial surgery.

In our operational definitions, the SSI was defined by the criteria of the Centers for Disease Control and Prevention (CDC),^25^ and we also included head CT scan features of intracranial SSI matching the clinical features suggestive of an infectious process included as follows.

The total hospital length was defined as the number of days from admission in the neurosurgery unit at discharge (or death), including the days of readmission.

The postoperative length of stay was defined as the number of days from the date of surgery of DSF at discharge (or death), including the days of readmission.

For the overall hospital outcomes at 3 months after the initial injury, since we recruited patients with TBI with a GCS >8, and our main outcome of interest was the occurrence of SSI, we did not use the Glasgow Outcome Scale for this variable since it does not capture complications of SSI. Indeed, the variable overall hospital outcome at 3 months after surgery was broadly divided into four groups in our study: initial improvement, prolonged hospital stay/care, reoperation, and death. The initial improvement at 3 months was defined by the resolution of the initial complaints to a healthy report of “no major concern” during the 3 months of review in the outpatient clinics; this included the uneventful healing of the surgical wound, relief of the neurological deficit, complete resolution of CSF, the correction of the deformity, and return to basic normal life activities. Complaints such as mild headache, dizziness, incisional mild pain or itching, scalp numbness, paresthesia, mood change, or any other drug side effects (constipation, etc.) were not considered as major complaints. The occurrence of a single event of post-traumatic seizure after surgery was not considered as part of the outcome assessment. Prolonged hospital stay/care was defined as a prolonged treatment for intravenous antibiotics, readmission for SSI, persistent CSF leak, or other major clinical complaints. Reoperation or death occurred either during the initial admission or readmission.

### Study procedures

All patients with TBI with suspicious skull fractures underwent initial care according to the Advanced Trauma Life Support protocol. They continued receiving routine trauma care such as analgesia, tetanus toxoid injection, maintenance i.v. fluids, and soft tissue suturing debridement. After obtaining a brain CT scan, patients with a diagnosis of DSF were identified by an active screening research team and were approached (or their attendants) for consent and recruitment into the study. Once recruited, patients were thereafter tracked by using a color-coded dot sticker on the file until the time of the DSF surgery. All the CDC guidelines for SSI prevention with aseptic protocol were strictly observed during and after the surgery as standard practice as well as the World Health Organization surgical safety checklist was observed. They all received perioperative intravenous antibiotic prophylaxis during anesthesia induction; the dosage of the drugs with a weight-adjusted dose for pediatric patients was given as follows: cefazolin 2 g with a repeat in 3–4 h of surgery, or ceftriaxone 2 g with a repeat in 3–4 h of surgery, or occasionally vancomycin 15 mg/kg, or ampicillin/sulbactam 2 g/1 g in continuation or substitution of the preoperative antibiotherapy.

The timing of surgery was recorded as the duration between the estimated time of injury and the surgical incision. From the surgical incision, all wounds were washed repeatedly with normal saline, regardless of the status of the initial soft tissue care or debridement from the A&E trauma team. A new incision was designed on the normal surrounding skin based on the need for surgery to avoid further manipulation of the nearby wound, if existent. The periosteum was highly preserved during the soft tissue dissection. As standard surgical procedures, beyond the outer edge of the depressed fragments and circumferentially, a craniotomy with a bone flap was drilled and milled, and a direct elevation of the depressed fragments was done. A piecemeal elevation was also observed in the case of comminuted DSF. Debridement and potential excision of small skull fragments was performed, followed by thorough washes using normal saline. Major skull fragments were primarily replaced depending on the neurosurgeon’s decision. There was evacuation of the underlying hematoma, if present, and also repair of the dural tear if found by primary closure or a harvested pericranial patch. During the surgical procedure, hemostasis was achieved with meticulous surgical dissection, cauterization, bone waxing, and a commonly used neurosurgical hemostat. At the end of the procedure, if necessary, an additional debridement of the wound margin of skin lacerations with copious irrigation and primary repair was performed, and closure with a sterile dressing with or without a drain was done. Patients were followed in the neurosurgical high dependency unit (HDU), intensive care unit (ICU), or ward during the postoperative period. Routine postoperative care was given to patients as requested in the postoperative instructions (analgesia, antibiotics, antiepileptic drugs, physiotherapy, etc.). All patients’ general and neurological assessment and wound care were recorded. Any occurrence of SSI infection or other types of morbidity was recorded during the hospital stay and in the outpatient clinics. A wound assessment was done during a change of dressing or clinical inspection by the attending clinician and the research assistant using the cranial SSI criteria of the CDC to diagnose the SSI^25^; clinical evidence of wound infections such as signs of inflammation, purulent discharge, or wound dehiscence (incomplete or complete) was recorded, and a swab of any wound discharge was taken for microbiological analysis of culture and sensitivity, as well as a complete blood count. Additional tests such as blood culture or urinalysis were recorded whenever possible to exclude other sources of infection. After the hospital discharge, even if the patients had no sign of SSI, they were followed up in the outpatients’ clinics within 10 weeks of suture or clip removal and every month (or at the time of occurrence of any major complaint) for the following 3 months for an eventual occurrence of SSI (superficial incisional infection, cranial osteomyelitis, epidural/subdural empyema, meningitis, or brain abscess). A follow-up brain CT scan was obtained if indicated for eventual intracranial infections.

### Data management and statistical analysis

Raw data were entered into an Excel spreadsheet, cleaned, and exported to STATA version 17 for analysis using both descriptive and inferential methods. Numerical data were summarized using median and interquartile range (IQR), whereas categorical data were summarized as frequencies and percentages. Continuous variables were compared across the categories of SSI and surgical timing using independent samples and Mann–Whitney test. Fisher’s exact test was used to check the difference between independent categorical variables with SSI and surgical timing. The bar charts, pie charts, and scatter plots were used to visualize the distribution of the outcome variables. The positive culture and sensitivity of results of patients’ wound samples were also analyzed, and the distribution of bacteria isolates was reported as proportions. Regression modeling was conducted using the modified Poisson model for the outcome variable SSI to adjust for the high prevalence (more than 10%), whereas the zero-truncated negative binomial model was used for the outcome variables postoperative hospital length of stay and total hospital length of stay to adjust for overdispersion and the absence of zero days. The bivariate analysis involved the inclusion of independent variables into the respective models one at a time, and only variables that had a *p* value <0.25 were considered for the multivariate regression modeling. Multivariate models for the outcome variables were logically developed using the forward stepwise approach, and only variables that had a *p* value <0.05 were considered significantly associated with the outcome. Sex and age variables were incorporated in the final predictors’ models regardless of their significance because they are known universal confounders in health studies. We reported the risk ratios for the modified Poisson model and the incidence rate ratios for the zero-truncated negative binomial model.

### Ethical consideration

This study received approval under the DESTINE Study from the Makerere University School of Medicine Research and Ethics Committee (Mak SOMREC) as SM-2020-7 and also at the Uganda National Council of Science and Technology (UNCST), registered as HS1284ES. This study respected all Good Clinical Practices used in clinical research by the Declaration of Helsinki. Patients or their legal next-of-kin (whenever applicable) consented to recruitment, and children above 8 years had a written assent in addition to their parental consent.

## Results

**FIG. 1.**
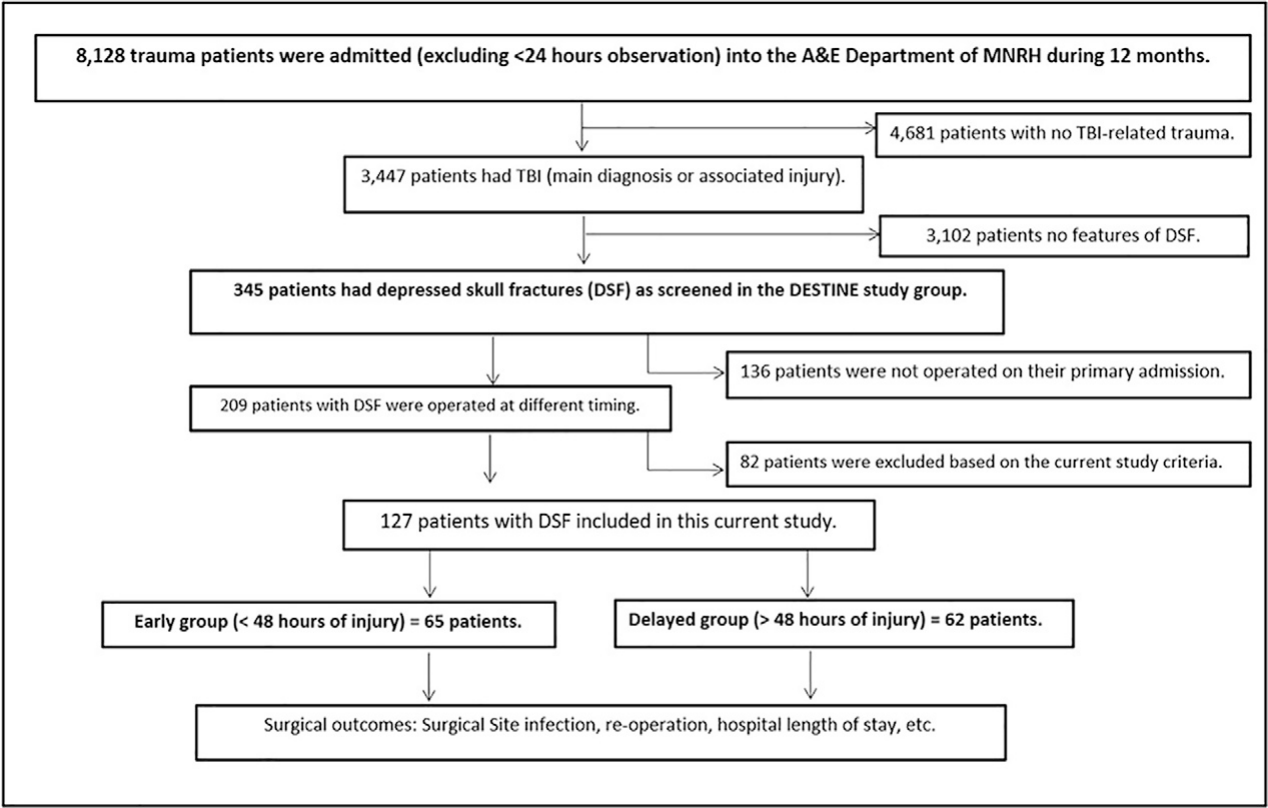
Patients’ flow chart.

We included a total of 127 operated patients with DSF in this study based on the study criteria as shown in the patients’ flow chart in Figure 1. This study was part of the DESTINE Study project that prospectively looked at the outcomes of DSF. The prevalence of DSF among admitted patients with TBI was 10%, with about 60.5% of them being managed operatively on the first admission. This prevalence is a proportion of 345 patients with TBI with the head CT diagnosis of DSF over a total of 3,447 patients with TBI admitted to the MNRH for at least 24 h in the neurosurgical wards during 1 year (March 2021–February 2022).

### Demographics and injury factors

The median age of our study participants was 24 years (IQR = 17–31; range = 2–61). The male–female sex ratio was 7.5 (Table 1). About 53.5% of them were victims of assaults, and half were injured by road traffic crashes (RTCs) either as passengers of a motorcycle (20.5%) or as pedestrians knocked by a speeding motorcycle (especially children under 8 years) or motor vehicle (18.9%); the remaining category was made up of victims of other mechanisms (7.1%) such as falling from high, falling heavy solid object over the head, or motor vehicle passengers. Most of the assault victims were either motorcycle taxi workers or their customers hit on the head with a metallic bar and brought to the nearby health facilities by the police patrol. A large number of them were referrals from other centers where there was a limited neurosurgical workforce.

**Table 1.** Distribution of Participants’ Demographics and Clinical Presentation by Surgical Timing

Variable	Overall	Surgical intervention timing group		Fisher’s exact
	*N* = 127 (row%)	≤48 h *N* (col%); 65 (51.2%)	>48 h *N* (col%); 62 (48.8%)	*p*-Value
Age (years), median (IQR); Mann–Whitney test	24 (17–31)	24 (18–29)	25 (17–32)	0.535
Sex				
Male	112 (88.2%)	59 (52.7%)	53 (47.3%)	0.417
Female	15 (11.8%)	6 (40.0%)	9 (60.0%)	
Mechanism of injury				
Assault	68 (53.5%)	36 (52.9%)	32 (47.1%)	0.467
Pedestrian knocked RTC	24 (18.9%)	13 (54.2%)	11 (45.8%)	
Passenger motorcycle RTC	26 (20.5%)	10 (38.5%)	16 (61.5%)	
Others	9 (7.1%)	6 (66.7%)	3 (33.3%)	
Long bone fracture (polytrauma)				
No	121 (95.3%)	63 (52.1%)	58 (47.9%)	0.433
Yes	6 (4.7%)	2 (33.3%)	4 (66.7%)	
Type of depressed skull fractures				
Simple	61 (48.0%)	32 (52.5%)	29 (47.5%)	0.860
Compound	66 (52.0%)	33 (50.0%)	33 (50.0%)	
Postresuscitation admission GCS				
9–13	34 (26.8%)	18 (52.9%)	16 (47.1%)	0.843
14–15	93 (73.2%)	47 (50.5%)	46 (49.5%)	
Neurological focal deficit				
No deficit	100 (78.7%)	51 (51.0%)	49 (49.0%)	0.555
Deficit	27 (21.3%)	14 (51.9%)	13 (48.1%)	
Post-traumatic seizures				
No	93 (73.3%)	49 (52.7%)	44 (47.3%)	0.689
Yes	34 (26.8%)	16 (47.1%)	18 (52.9%)	
Preoperative ASA classification				
Class 1	10 (7.9 %)	4 (40.0%)	6 (60.0%)	0.085
Class 2	98 (77.2%)	47 (48.0%)	51 (52.0%)	
Class 3	19 (14.9%)	14 (73.7%)	5 (26.3%)	
Preoperative hemoglobin (g/dL)				
>12	72 (56.7%)	40 (55.6%)	32 (44.4%)	0.544
10–11	37 (29.1%)	17 (45.9%)	20 (54.1%)	
<10	18 (14.2%)	8 (44.4%)	10 (55.6%)	

ASA, American Society of Anesthesiologists; GCS, Glasgow Coma Scale; IQR, interquartile range; RTC, road traffic crashes.

### Clinical presentation and brain imaging

About 73.2% had a postresuscitation GCS of 14 and 15. There were 21.3% of patients who presented with a focal neurological deficit at admission (hemiparesis, monoparesis, and cranial nerve palsy), and five patients had aphasia. About 26.8% of them have a history of post-traumatic seizures (immediate or early) almost as generalized tonic–clonic convulsions. Active CSF rhinorrhea was observed in the involvement of the anterior skull base of the frontal DSF in 4 (3.15%) patients, associated with maxilla–facial fractures.

**FIG. 2.**
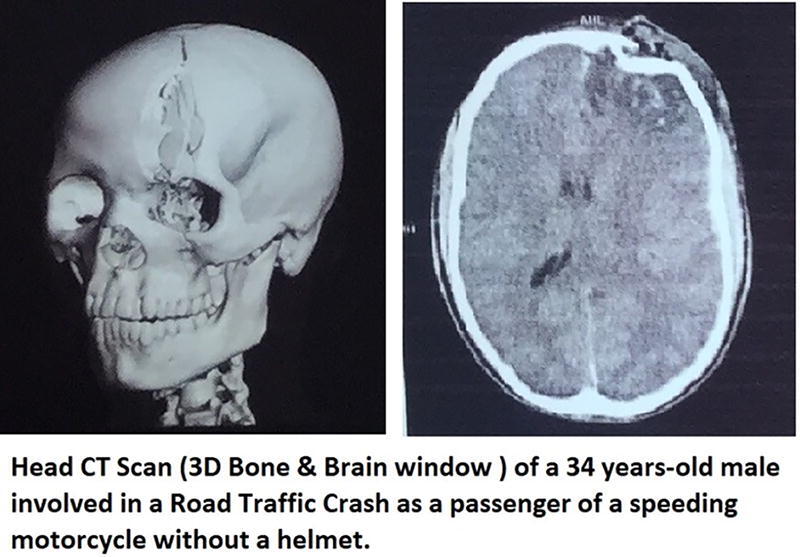
Illustration of head CT scan of a case of depressed skull fracture involving the frontal air sinus and orbital rim. CT, computed tomography.

About 4.7% of those patients presented with associated injuries (long bone fractures). All patients had at least one brain CT scan following admission, but there was a noticeable delay in obtaining brain CT imaging among patients referred from other health facilities. The frontal bone was the most frequently involved (59%), followed by the parietal bone (48.8%). The brain CT scan frequently shows underlying cerebral contusion (84.2%), underlying extra-axial hemorrhage (28.4%), air sinus involvement (15.0%), pneumocranium (9.5%), basal cisterns open (60.6%), and midline shift <5mm (8.7%), as shown in Table 2. About 8 (11.6%) of them had intraparenchymal bleeding (intracerebral hemorrhage). Figure 2 illustrates an example of a head CT scan of a 34-year-old male with a frontal DSF with an underlying contusion. Most of the study patients had a hemoglobin rate above 12 g/dL before surgery (85.8%), and the preoperative ASA class 2 was the most common type (77.2%) as assessed by the anesthesiologists on duty. All patients tested COVID-19 negative with the systematic nasopharyngeal swab polymerase chain reaction (PCR) testing.

**Table 2.** Distribution of Patients’ Head CT Findings, Management, and Outcomes by the Surgical Timing of DSF

Variable	Overall	Surgical intervention timing group		Fisher’s exact
	*N* = 127 (row%)	≤48 h *N*(col%); 65 (51.2%)	>48 h *N*(col%); 62 (48.8%)	*p*-Value
Location of DSF				
Frontal	46 (36.2%)	23 (50.0%)	23 (50.0%)	0.775
Frontal parietal	29 (22.8%)	14 (48.3%)	15 (51.7%)	
Frontal temporal	5 (3.9%)	4 (80.0%)	1 (20.0%)	
Parietal	33 (26.0%)	16 (48.5%)	17 (51.5%)	
Temporal	14 (11.0%)	8 (57.1%)	6 (42.9%)	
Extra-axial hemorrhage				
No	91 (71.7%)	46 (50.5%)	45 (49.5%)	0.846
Yes	36 (28.4%)	19 (52.8%)	17 (47.2%)	
Presence of underlying cerebral contusion				
No	20 (15.8%)	12 (60.0%)	8 (40.0%)	0.469
Yes	107 (84.2%)	53 (49.5%)	54 (50.5%)	
Pneumocranium				
No	115 (91.3%)	58 (50.4%)	57 (49.6%)	0.764
Yes	12 (9.5%)	7 (58.3%)	5 (41.7%)	
Air sinus involvement				
No	108 (85.0%)	60 (55.6%)	48 (44.4%)	**0.025**
Yes	19 (15.0%)	5 (26.3%)	14 (73.7%)	
Basal cisterns in head CT finding				
Absent	10 (7.9%)	2 (20.0%)	8 (80.0%)	0.076
Open	77 (60.6%)	39 (50.6%)	38 (49.4%)	
Compressed	40 (31.5%)	24 (60.0%)	16 (40.0%)	
Midline shift >5 mm				
No	116 (91.3%)	60 (51.7%)	56 (48.3%)	0.760
Yes	11 (8.7%)	5 (45.6%)	6 (54.5%)	
Duration of Anesthesia (minutes), median (IQR); Mann–Whitney test	120 (97–155)	120 (95–145)	125 (104–165)	0.057
Duration of surgery procedure (minutes), median (IQR); Mann–Whitney test	80 (60–100)	75 (60–90)	88 (60–115)	**0.0194**
Intraop. finding of underlying dural tear				
No	63 (49.6%)	33 (52.4%)	30 (47.6%)	0.860
Yes	64 (50.4%)	32 (50.0%)	32 (50.0%)	
Surgical site infection				
No	103 (81.1%)	59 (57.3%)	44 (42.7%)	**0.006**
Yes	24 (18.9%)	6 (25.0%)	18 (75.0%)	
Hospital outcomes after the initial surgery				
Improved	102 (80.3%)	58 (56.9%)	44 (43.1%)	**0.050**
Reoperated	7 (5.5%)	2 (28.6%)	5 (71.4%)	
Died	2 (1.6%)	0 (0%)	2 (100%)	
Prolonged stay	16 (12.6%)	5 (31.3%)	11 (68.8%)	
Postoperative length of stay in neurosurgery (days) median (IQR); Mann–Whitney test *p* value	3 (3–5)	3 (3–5)	4 (3–6)	0.1443
Total length of hospital stay (days) median (IQR); Mann–Whitney test *p* value	6 (4–8)	5 (4–9)	8 (6–12)	**<0.001**

Bold value signifies *p*-value <0.05.

DSF, depressed skull fracture; IQR, interquartile range.

### Management

Every patient received routine trauma care from admission (100%). Medical management administered to those patients consisted of analgesics (100%), antiepileptic drugs (100%), tranexamic acid (67.9%), and preoperative osmotherapy (37.8%). Prophylactic intraoperative antibiotics were given to all patients (100%). There were 48.0% clinically classified simple versus 52.0% compound DSF. There were 51.2% who were operated on before 48 h of injury (early group) and 48.8% who were operated on after 48 h (delayed). All (100%) of the neurosurgical operations were done under general anesthesia, and almost all were performed by an attending neurosurgeon leading the surgical team or supervising a senior neurosurgery resident on the operating table. The median duration of anesthesia was 120 (IQR: 97–155) min, and the mean duration of the surgical procedure was 80 (IQR: 60–90) min (Table 2).

Surgical management consisted of a craniotomy for debridement and elevation (100%) and additional hematoma evacuation. About 50.4% of patients had an intraoperative finding of dural tear, and subsequent duroplasty was performed. All (100%) of the patients had routine postoperative care in the HDU/ICU and ward for 24 h. They all had routine clinical reviews and wound care in postoperative care.

### Surgical outcomes and effects of the surgical timing

**FIG. 3.**
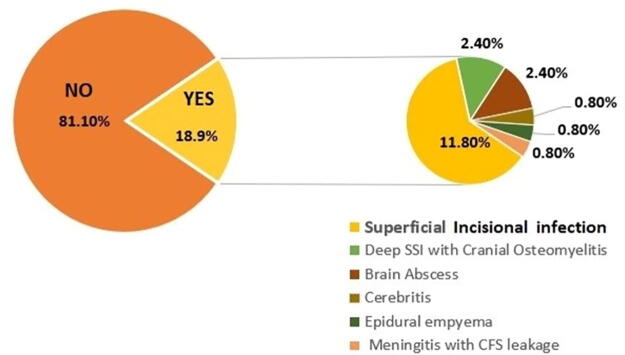
Proportion and patterns of surgical site infections among patients with depressed skull fractures.

There were 24 (18.9%) who developed SSI; the most common type of SSI was the superficial incisional infection (soft tissue infection) (Fig. 3), and it was mainly diagnosed within the first 7 days of the postoperative period. There were seven cases of readmission and reoperations for cranial SSI for either osteomyelitis debridement or frank pus drainage diagnosed beyond 14 days of surgery. Intracranial infection was diagnosed later after obtaining a brain CT scan from the clinical features in the outpatients’ clinics or at readmission.

**FIG. 4.**
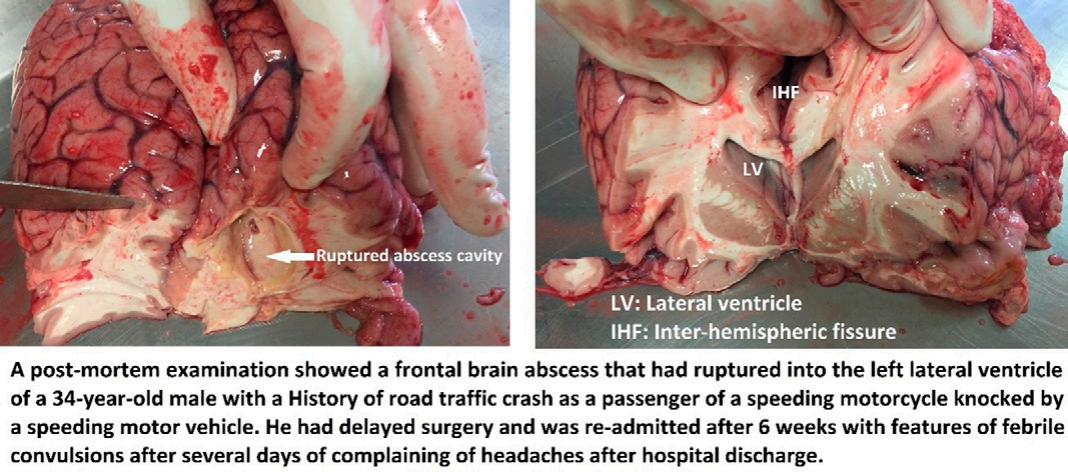
Illustration of a postmortem examination of a patient (same in Fig. 3) who died with a brain abscess.

The overall postoperative length of stay in neurosurgery was 3 (IQR = 3–5) days. The overall median length of stay in the hospital was 6 (IQR: 4–8) days. The mortality was 2/24 (8.33%) among patients with an occurrence of cranial SSI, mainly associated with frontal brain abscess and cerebritis in surgery done after 48 hours; an illustration of a postmortem tissue examination of a patient with a brain abscess is shown in Figure 4.

**FIG. 5.**
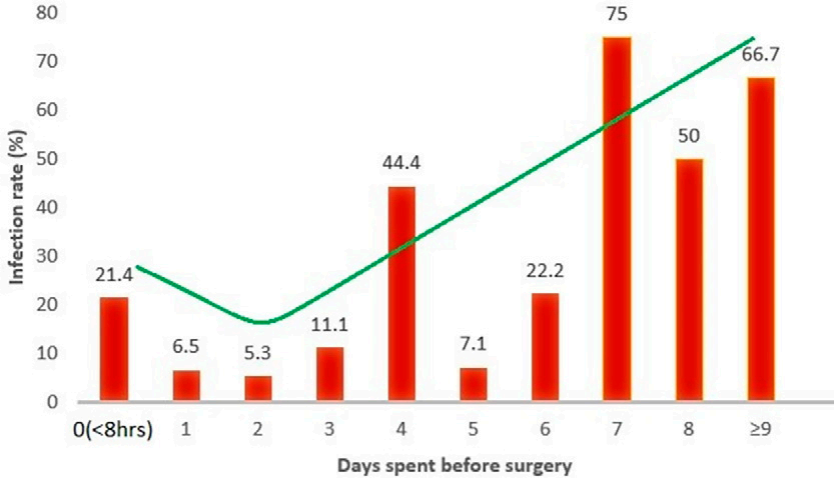
Bar graph showing the surgical site infection rate against the days spent before surgery.

**FIG. 6.**
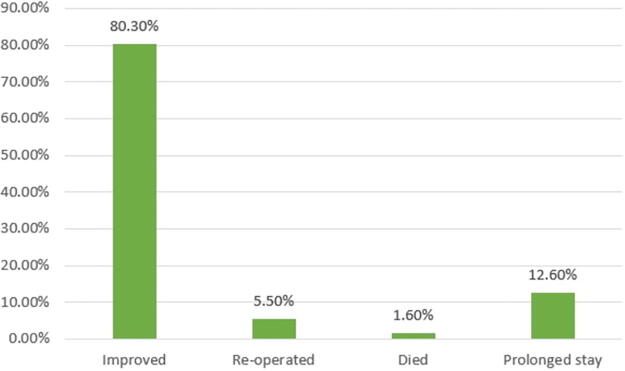
Bar graph showing the overall hospital outcomes after the initial surgery.

The delayed group had an increased incidence (75% vs. 25%) of SSI within 3 months in comparison to the early group (*p* = 0.006); additionally, the delayed group had a longer median postoperative hospital stay of 8 (IQR = 6–12) days versus 5 (IQR = 4–9) days when compared with the early group (Mann–Whitney test *p* < 0.001), and a higher rate of reoperation of 71.4% versus 28.6% in the delayed group compared with the early group was reported (Fisher’s exact test *p* = 0.05). When the early group was stratified into two groups (before 24 h and 24–48 h) in the subanalysis, we found that there was no statistically significant difference in the risk of SSI and hospital length of stay among the two groups (Supplementary Tables S1 and S2). The trend of incidence of infection was slightly higher for surgery done urgently before 8 h of injury, then decreased for those done in the next 48 h of injury, and then steeply rose higher every day from 72 h and beyond (Fig. 5). In general, most patients in this study had a good recovery or improvement rate (80.3%) after the initial surgery of DSF within 3 months (Fig. 6). About two patients still had persistent upper limb monoparesis and facial nerve palsy, respectively, but with an improvement.

### Bacteriological findings

Among the 24 (18.9%) who developed SSI, there were 18 pus swab samples from patients that had shown bacteriologically evidenced SSI after culture and sensitivity. Pus swab samples from 2 patients had no bacterial growth after 72 hours of microbiological culture and antibiotics sensitivity; 4 patients had obvious clinical-radiological features of deep intracranial SSI without bacterial sampling for microbiology. Gram-negative microorganisms constituted the highest number of isolates with 11/17 (64.7%), of which *Escherichia coli* species constituted 4/17 (23.5%) and *Klebsiella pneumoniae* constituted 4/11 (23.5%). Among the Gram-positive bacteria, 4/17 (23.5%) were *Enterococcus* species, and 2/17 (11.8%) were *Staphylococcus aureus*. Gram-positive isolates showed resistance to most of the commonly prescribed antibiotics. The two cases of mortality due to intracranial SSI were attributed to poly-microorganism infections associated with *E. coli*; this was evidenced by a CSF sample of one patient and from a postmortem examination of an abscess isolate from another patient on a recent death (shown in Fig. 3).

### Predictors of SSI

A bivariate analysis between surgical timing (early or delayed surgery) and all other parameters was performed using the Fisher’s exact test for categorical variables and the Mann–Whitney test for continuous variables. The presence of air sinus involvement with the DSF was found to be associated with surgical timing (Fisher’s exact test *p* = 0.025), whereas a longer duration of anesthesia (Mann–Whitney test *p* = 0.057) and a longer duration of the surgical procedure (Mann–Whitney test *p* = 0.0194) were observed among patients whose surgery was performed after 48 h, as shown in Table 2. When performing bivariate analysis between the group of SSI and no SSI, the parameters of delayed surgical timing (Fisher’s exact test *p* = 0.006), the clinical type of compound DSF (Fisher’s exact test *p* = 0.013), the frontal location of DSF (Fisher’s exact test *p* = 0.015), the presence of pneumocranium in head CT scan (Fisher’s exact test *p* = 0.011), the involvement of air sinus in the DSF (Fisher’s exact test *p* < 0.001), and the intraoperative finding of a dural tear (Fisher’s exact test *p* = 0.04) were statistically significant (Table 3).

**Table 3.** Predicting Models of the Effect of the Surgical Timing on the Occurrence of Surgical Site Infection

Variable	Surgical site infection		Fisher’s exact	Model estimates	
	Yes: *N*(col%)	No: *N*(col%)	*p*-Value	Unadjusted RR (95% CI)	Adjusted RR (95% CI)
Surgical timing group					
Surgical intervention ≤48 hours	6 (9.2%)	59 (90.8%)	**0.006**	**1**	**1**
Surgical intervention >48 hours	18 (29.0%)	44 (71.0%)		3.15 (1.33–7.43)^***^	2.79 (1.25–6.22)^*^
Age (years)				1.02 (1.00–1.05)	1.01 (0.99–1.04)
Sex					
Female	2 (13.3%)	13 (86.7%)	0.735	1	1
Male	22 (19.6%)	90 (80.4%)		1.47 (0.38–5.68)	1.14 (0.44–2.95)
Mechanism of injury					
Assault	11 (16.2%)	57 (83.8%)	0.417	1	
Pedestrian knocked RTC	4 (16.7%)	20 (83.3%)		1.03 (0.36–2.94)	
passenger motorcycle RTC	8 (30.8%)	18 (69.2%)		1.90 (0.86–4.21)	
Others	1 (11.1%)	8 (88.9%)		0.69 (0.10–4.75)	
Long bone fracture (polytrauma)					
No	21 (17.4%)	100 (82.6%)	0.081	**1**	
Yes	3 (50%)	3 (50%)		2.88 (1.18–7.04)^*^	
Type of DSF					
Simple	6 (98.4%)	55 (90.2%)	**0.013**	**1**	
Compound	18 (27.3%)	48 (72.7%)		2.77 (1.17–6.55)^*^	
Postresuscitation admission GCS					
9–13	10 (29.4%)	24 (70.6%)	0.078	1	**1**
14–15	14 (15.1%)	79 (84.9%)		1.95 (0.96–3.99)	1.96 (1.02–3.77)^*^
Neurological focal deficit					
No deficit	18 (18.0%)	82 (82.0%)	0.590	1	
Deficit	6 (22.2%)	21 (77.8%)		1.23 (0.54–2.81)	
Post-traumatic seizures					
No	18 (19.4%)	75 (80.6%)	0.525	1	
Yes	6 (17.6%)	28 (82.4%)		0.91 (0.39–2.11)	
Preoperative hemoglobin					
>12	11 (15.3%)	61 (84.7%)	0.229	1	
10–11	7 (18.9%)	30 (81.1%		1.24 (0.52–2.94)	
<10	6 (33.3%)	12 (66.7%)		2.18 (0.93–5.12)	
Location of DSF					
Frontal	15 (32.6%)	31 (67.4%)	**0.015**	**1**	
Frontal parietal	1 (3.45%)	28 (96.6%)		**0.11 (0.01–0.76)** ^*^	
Frontal temporal	1 (20.0%)	4 (80.0%)		0.61 (0.10–3.74)	
Parietal	6 (18.2%)	27 (81.8%)		0.56 (0.24–1.29)	
Temporal	1 (7.1%)	13 (92.9%)		0.22 (0.03–1.53)	
Extra-axial hemorrhage					
No	71 (78.0%)	20 (22.0%)	0.211	1	
Yes	32 (88.9%)	4 (11.1%)		0.51 (0.18–1.38)	
Pneumocranium					
No	18 (15.7%)	97 (84.3%)	**0.011**	**1**	**1**
Yes	6 (50.0%)	6 (50.0%)		3.73 (1.93–7.21)^**^	2.84 (1.50–5.36)^**^
Air sinus involvement					
No	14 (13.0%)	94 (87.0%)	**0.000**	1	**1**
Yes	10 (52.6%)	9 (47.4%)		4.06 (2.12–7.79)	2.91 (1.55–5,47)^**^
Basal cisterns head CT finding					
Absent	2 (20.0%)	8 (80.0%)	0.773	1	
Open	16 (20.8%)	61 (79.2%)		1.04 (0.28–3.89)	
Compressed	6 (15.0%)	34 (85.0%)		0.75 (0.18–3.19)	
Midline shift >5 mm					
No	22 (19.0%)	94 (81.0%)	0.656	1	
Yes	2 (18.2%)	9 (81.8%)		0.96 (0.26–3.57)	
Preoperat. ASA classification					
Class 1	8 (80.0%)	2 (20.0%)	1.000	1	
Class 2	79 (80.6%)	19 (19.4%)		0.97 (0.26–3.59)	
Class 3	16 (84.2%)	3 (15.8%)		0.79 (0.16–4.00)	
Intraop finding of dural tear					
No	7 (11.1%)	56 (88.9%)	**0.040**	1	
Yes	17 (26.6%)	47 (73.4%)		**2.39 (1.06–5.38)** ^*^	
Duration of anesthesia	—	—		1.00 (0.99–1.01)	
Duration of surgical procedure	—	—		1.00 (0.99–1.01)	

^*^ *p* < 0.05.

^**^ *p* < 0.01.

^***^ *p* < 0.001.

Bold value signifies p-value <0.05.

ASA, American Society of Anesthesiologists; CI, confidence interval; CT, computed tomography; DSF, depressed skull fracture; GCS, Glasgow Coma Scale; RR, risk ratio; RTC, road traffic crashes.

At bivariate analysis using the modified Poisson model, we found that delayed surgery (above 48 h) of DSF (unadjusted risk ratio [URR], 3.15; 95% confidence interval [CI], 1.33–7.43), the presence of pneumocranium (URR, 3.73; 95% CI: 1.93–7.23), associated fracture of long bones (URR, 2.88; 95% CI: 1.18–7.04), the clinical type of compound DSF (URR, 2.77; 95% CI: 1.17–6.55), and the intraoperative finding of a dural tear (URR, 2.39; 95% CI: 1.06–5.38) were, respectively, associated with SSI (Table 3).

**FIG. 7.**
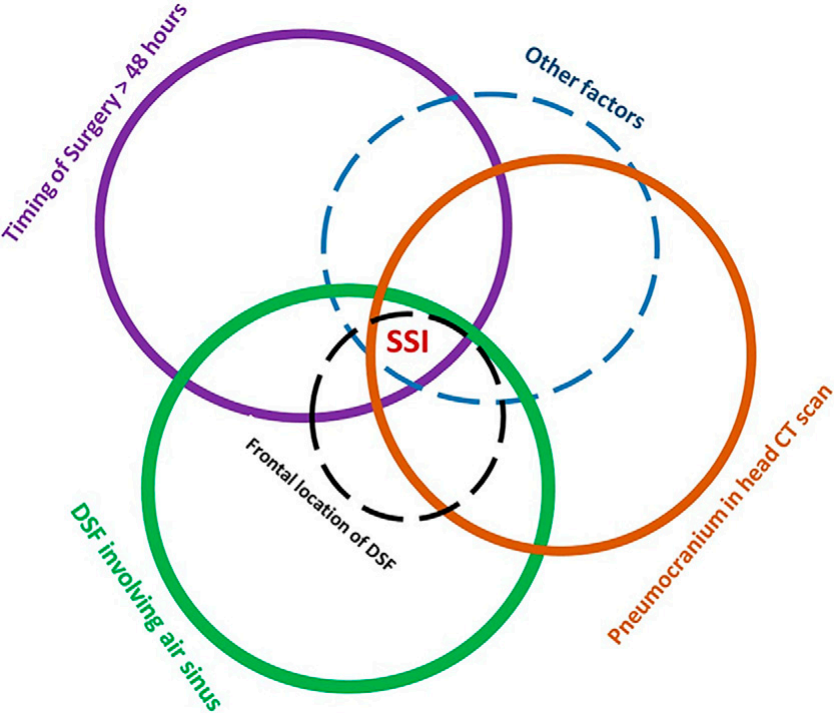
Graphical modeling with size-weighted preoperative predicting factors (in circles) of surgical site infections following surgical intervention of depressed skull fractures.

When performing multivariate analysis between the group of SSI and no SSI, the parameters delayed surgical timing (adjusted risk ratio [ARR], 2.79; 95% CI: 1.25–6.22), the presence of pneumocranium (ARR, 2.84; 95% CI: 1.50–5.36), and involvement of air sinus (ARR, 2.91; 95% CI: 1.55–5.47) were associated with a more than a 2.5-fold increase in the rate of SSI when compared, respectively, with the early surgery, the absence of pneumocranium, and no involvement of air sinus. The parameter postresuscitation GCS 9–13 (ARR, 1.96; 95% CI: 1.02–3.77) was predicting a 96% increase in risk of SSI when compared with those with postresuscitation GCS above 13 (Table 3). A designed graphical model with size-weighted predictors of SSI in patients with DSF shows an increased risk of SSI when the different independent predicting circles intercross (Fig. 7).

### Effect of the surgical timing on secondary outcomes of the study

The effects of the parameters on the postoperative hospital length of stay were modeled using the zero-truncated negative binomial model to adjust for overdispersion in the data and also due to the absence of zero counts in the data. At bivariate analysis, we found that the delayed surgery group (unadjusted incidence rate ratio [UIRR], 1.30; 95% CI: 1.00–1.71), long bone fracture (UIRR,1.36; 95% CI: 1.04–1.78), type of compound DSF (UIRR, 1.55; 95% CI: 1.21–1.98), intraoperative finding of dural tear (UIRR, 1.55; 95% CI: 1.21–1.99), and pneumocranium (UIRR, 1.58; 95% CI: 1.03–2.42) were associated with the postoperative hospital length of stay after admission for DSF (Table 4).

**Table 4.** Association Between Surgical Timing and Postoperative Hospital Length of Stay

Variable	Model estimates	
	Unadjusted IRR (95% CI)	Adjusted IRR (95% CI)
Surgical timing group		
Early (≤48 h)	1	1
Delayed (>48 h)	1.31 (1.00–1.71)	1.28 (1.01–1.63)^*^
Age (years)	1.01 (0.99–1.02)	1.01 (0.99–1.02)
Sex		
Female	1	**1**
Male	1.26 (0.84–1.89)	**1.23 (0.86–1.75)**
Mechanism of injury		
Assault	1	
Pedestrian knocked RTC	1.18 (0.75–1.86)	
passenger motorcycle RTC	1.14 (0.81–1.62)	
Others	0.91 (0.67–1.22)	
Long bone fracture (polytrauma)		
No	1	
Yes	1.36 (1.04–1.78)^*^	
Type of DSF		
Simple	1	**1**
Compound	1.55 (1.21–1.98)^***^	1.43 (1.13–1.80)^**^
Post resuscitation admission GCS		
14–15	1	
9–13	1.25 (0.97–1.60)	
Neurological focal deficit		
No deficit	1	
Deficit	1.11 (0.83–1.48)	
Post-traumatic seizures		
No	1	
Yes	0.98 (0.69–1.39)	
ASA classification		
Class 1	1	
Class 2	0.65 (0.35–1.19)	
Class 3	0.72 (0.38–1.37)	
Preoperative hemoglobin		
>12	1	
10–11	1.08 (0.78–1.48)	
<10	1.07 (0.78–1.48)	
Location of DSF		
Frontal	1	
Frontal parietal	0.76 (0.57–1.02)	
Frontal temporal	1.16 (0.82–1.66)	
Parietal	0.98 (0.64–1.50)	
Temporal	0.82 (0.61–1.09)	
Extra-axial hemorrhage		
No	1	
Yes	0.73 (0.58–0.92)^**^	
Presence of cerebral contusion		
No	1	**1**
Yes	1.61 (1.30–1.99)^***^	1.35 (1.09–1.68)^**^
Pneumocranium		
No	1	
Yes	1.58 (1.03–2.42)^*^	
Air sinus involvement in DSF		
No	1	
Yes	1.39 (0.97–1.98)	
Basal cisterns in radiological finding		
Absent	1	
Open	1.20 (0.88–1.63)	
Compressed	1.07 (0.81–1.40)	
Midline shift >5 mm		
No	1	
YES	0.98 (0.76–1.26)	
Intraop. finding of dural tear		
No	1	
Yes	1.55 (1.21–1.99)^**^	
Duration of anesthesia	1.00 (.99–1.00)	
Duration of surgical procedure	1.00 (1.00–1.01)	

^*^ *p* < 0.05.

^**^ *p* < 0.01.

^***^ *p* < 0.001.

Bold value signifies *p*-value <0.05.

ASA, American Society of Anesthesiologists; CI, confidence interval; DSF, depressed skull fracture; GCS, Glasgow Coma Scale; IRR, incidence rate ratios; RTC, road traffic crashes.

At multivariate analysis of postoperative duration, we found that delayed surgical timing (adjusted incidence rate ratio [AIRR] 1.28; 95% CI: 1.01–1.63), the presence of underlying cerebral contusion (AIRR, 1.35; 95% CI: 1.09–1.68), and the compound type of DSF (AIRR, 1.43; 95% CI: 1.13–1.80) were, respectively, associated with a 25%, 35%, and 43% increase of postoperative hospital length of stay when compared with their reference categories (Table 4).

**FIG. 8.**
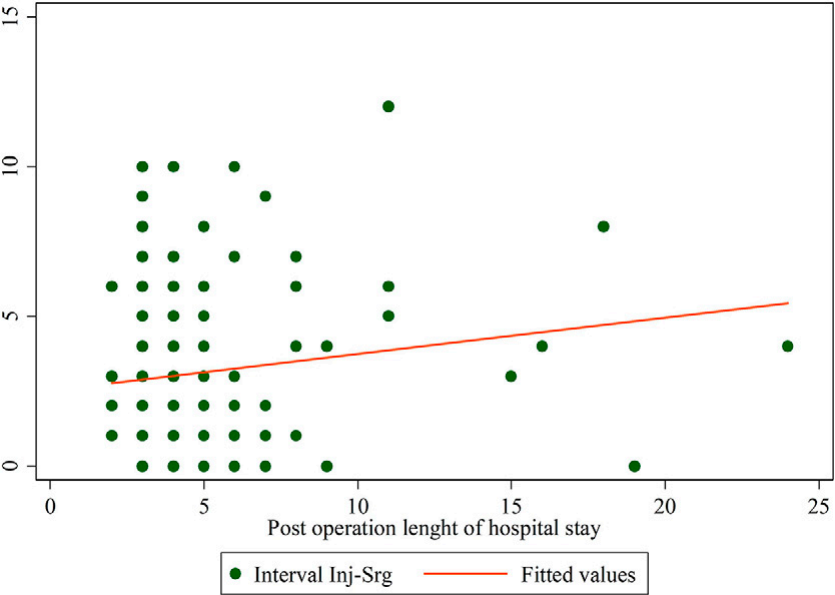
Scatter plot for the relationship between days spent before surgery and postoperative hospital length of stay (including readmission).

There was a weak association between the timing of surgery and the postoperative length of stay (including the readmission hospital stay), with a correlation coefficient of 0.16 (Fig. 8).

At bivariate analysis of predictors of total hospital length of stay, we found that the delayed surgery group (UIRR, 1.90; 95% CI: 1.55–2.32), long bone fracture (UIRR, 1.44; 95% CI: 1.09–1.89), type of compound DSF (UIRR, 1.41; 95% CI: 1.13–1.77), intraoperative finding of dural tear (UIRR, 1.36; 95% CI: 1.08–1.71), and air sinus involvement (UIRR, 1.42; 95% CI: 1.08–1.88) were associated with the total hospital length of stay from the admission (Table 5).

**Table 5.** Association Between Surgical Timing and Total Length of Hospital Stay

Variable	Model estimates	
	Unadjusted IRR (95% CI)	Adjusted IRR (95% CI)
Surgical timing group		
Early (≤48 h)	1	1
Delayed (>48 hours)	1.90 (1.55–2.32)^***^	1.86 (1.55–2.25)^***^
Age (years)	1.01 (0.99–1.02)	1.00 (1.00–1.01)
Sex		
Female	1	1
Male	1.09 (0.72–1.66)	1.10 (0.80–1.52)
Mechanism of injury		
Assault	1	
Pedestrian knocked RTC	1.11 (0.77–1.58)	
passenger motorcycle RTC	1.11 (0.83–1.49)	
Others	1.02 (0.68–1.52)	
Long bone fracture (polytrauma)		
No	1	1
Yes	1.44 (1.09–1.89)^*^	1.29 (1.03–1.61)^*^
Type of DSF		
Simple	1	1
Compound	1.41 (1.13–1.77)^**^	1.37 (1.14–1.64)^**^
Postresuscitation admission GCS		
9–13	1	
14–15	0.78 (0.59–1.02)	
Neurological focal deficit		
No deficit	1	
Deficit	1.12 (0.87–1.45)	
Post-traumatic seizures		
No	1	
Yes	0.94 (0.72–1.22)	
Preoperative hemoglobin		
>12	1	
10–11	1.20 (0.92–1.55)	
<10	1.39 (0.91–2.12)	
Location of DSF		
Frontal	1	
Frontal parietal	0.80 (0.62–1.04)	
Frontal temporal	0.86 (0.67–1.12)	
Parietal	0.98 (0.71–1.35)	
Temporal	1.00 (0.56–1.78)	
Pneumocranium in head CT		
No	1	
Yes	1.17 (0.88–1.57)	
Air sinus involvement		
No	1	
Yes	1.42 (1.08–1.88)^*^	
Basal cisterns head CT finding		
Absent	1	
Open	0.97 (0.72–1.29)	
Compressed	0.90 (0.64–1.26)	
Midline shift >5 mm		
No	1	
Yes	1.31 (0.74–2.31)	
Intraoperative finding of dural tear		
No	1	
Yes	1.36 (1.08–1.71)^**^	

^*^p < 0.05.

^**^ *p* < 0.01.

^***^ *p* < 0.001.

Bold value signifies p-value <0.05.

ASA, American Society of Anesthesiologists; CI, confidence interval; DSF, depressed skull fracture; GCS, Glasgow Coma Scale; IRR, incidence rate ratios; RTC, road traffic crashes.

At multivariate analysis of total duration of hospital stay, we found that delayed surgical timing (AIRR, 1.86; 95% CI: 1.55–2.25), the compound type of DSF (AIRR, 1.37; 95% CI: 1.14–1.64), and the presence of associated long bone fracture (AIRR, 1.29; 95% CI: 1.03–1.61) were, respectively, associated with a 86%, 37%, and 29% increase of the total length of hospital stay when compared with their reference categories (Table 5).

## Discussion

This study was set up to compare the risk of developing SSI within 3 months between surgery of DSF done before and after 48 h of injury in patients with nonsevere TBI and the preoperative clinical-radiological predictors of the occurrence of cranial SSI. We reported a prevalence of DSF of 10% among admitted patients with TBI. The prevalence of DSF in our study is almost the same as reported in a recent descriptive study on DSF, about 11.3% among TBI.^26^

### Demographics and injury factors

The two groups of interest (early versus delayed surgery) had similar demographics (age, sex, and other injury factors). Most of the study participants were young male adults, as reported in other literature, with a median age of 24 years. This was similar to the studies on DSF in the literature on different continents.^27–30^ We found that 53.5% were victims of assaults, such as in many other similar studies on DSF^27,31^; this is mainly done by the criminal phenomenon related to boda-boda (motorcycle) taxi transportation in Uganda, where the victims, motorcycle taxi men or their customers, are hit on the head by a metallic bar or hammer late at night or before the sunrise by unknown thieves and then robbed after. This phenomenon has overtaken the number of victims with TBI with DSF in Uganda in comparison with RTC. Most of those trauma victims are brought by the police patrol, as reported in another study in Uganda.^23^ Other studies found that RTC was the main mechanism leading to the DSF,^28,30^ although, in Pakistan, another study pointed out fall as the commonest mechanism up to 52% of DSF,^32^ surely due to other local circumstances of injury. This may be due to the exclusion criteria of our study, where most of the victims of RTC had severe TBI. We also reported a higher proportion (18.9%) of pediatric DSF that has occurred as pedestrians knocked by a speeding motorcycle or motor vehicle by mechanism. This unusual situation is mainly due to an increasingly complex road environment with unattended pediatric road users combined with the increasing motorization of motorcycle taxi business (boda-boda) in Uganda and East Africa in general, where road traffic regulations are lacking safety observance.^33,34^

### Clinical presentations and brain imaging

Here again, the two groups of interest (early vs. delayed surgery) had quite similar features. Patients in our study were admitted with a postresuscitation GCS of 14–15 in 73.2%, as found in other studies.^27,30,35^ That is a group of patients with TBI where the emergency neurosurgical procedure is often delayed in SSA in the absence of other additional indications of surgery for DSF and also a delayed presentation from underestimating the magnitude of injury when the patient has regained consciousness. The retrospective study from Ethiopia reported a mean of 15 h from injury to admission.^27^ A few of them presented with associated long bone fractures, certainly associated with the mechanism of being knocked by a speeding vehicle (RTC), where both the point of impact with the vehicle (frequently the limb) and the one at the projected organ on the hard surface (frequently the head) contributed to multiple fracture injuries. About 21.3% of patients had hemiparesis, monoparesis, and/or cranial nerve palsy at admission. In our study, 26.8% had at least one episode of a post-traumatic seizure (immediate or early). This figure was higher than the study done by Oboth *et al.* where they excluded the pediatric population and also included nonoperative patients.^26^ Another study reported 12.3% for early post-traumatic seizure in the hospital during 1 week on 73 patients with surgically treated DSFs from 1993 to 1998^29^; in our study, we excluded patients with large extra-axial hemorrhage considered as the primary indication of surgery, and having more patients with contusion, including both immediate and early seizure recorded actively from the injury site up to 1 week, increases the detection of seizure events. As reported in many studies, the frontal bone is the most frequently involved, followed by the parietal bone,^26,27,30,35,36^ yet others report the reverse.^37,38^ In brain CT findings, underlying intracranial injuries are known to be frequently associated with DSFs as direct brain damage (cerebral contusions), vascular damage (extra-axial hematoma), and so forth.^3–5,39^ Underlying cerebral contusion was the most frequent (84.2%), followed by underlying extra-axial hemorrhage (28.4%); this higher prevalence of brain contusion is also due to the same exclusion criteria of large extra-axial hemorrhage and penetrating mechanism. The air-sinus involvement was 15.0%, especially with frontal DSF. Frontal air sinus involvement induces a traumatic cranialization of those pneumatized cavities in continuation with the nasal cavity; this implies that the colonized sinus mucosa becomes a potential source of nearby contamination and SSI. The pneumocranium, an indirect evidence of CSF leakage (active or not), was found in 9.5%; in contrast to another study, it is around 23%^30^; this prevalence is relatively lower because we excluded the penetrating mechanism that usually results in a higher incidence of pneumocranium with a higher rate of infections.^40^ Some of these key studies on DSF were previously conducted before the CT scan era and, thus, could not correlate the results with the underlying intracranial injuries. Our study has included several head CT parameters that could contribute to the occurrence of SSI. Most of our participants were hemodynamically stable, with a higher GCS score perioperatively with no features of significant brain edema on the CT scan, a presurgical hemoglobin rate above 12 g/dL (85.8%), and an ASA classification frequently below 3 (85.2%), showing that they were relatively fit for general anesthesia without other systemic concerns. Most studies done retrospectively in DSF had little details in exclusion criteria, which assumes that they included also severely ill patients with systemic disease that could have influenced largely the surgical outcomes independently of the surgical pathology in contrast to our study.^29^

### Management

Our patients had almost received standardized acute care in perioperative management, consisting mainly of resuscitation, prophylaxis antibiotics, antiepileptic treatment, and other treatments. Prophylactic intraoperative antibiotics were observed as recommended by most of the SSI prevention bundles.^25^ Indeed, we also included both clinically classified simple (48.0%) and compound DSF (52.0%). Most studies reported more than four decades ago did not find an equipoise on the rate of the SSI^41–43^ regarding simple versus compound, but later studies did, maybe because the operational definition of compound DSF is always inconsistent in the literature of open head injury versus closed. In our study, the compound type was relatively associated with a higher incidence of SSI in comparison to simple DSF at univariate analysis. The duration of anesthesia was 40 min longer than the actual procedure, basically because our patients were hemodynamically stable with GCS above 8, and we included the pediatric population that tends to consume anesthesia time to be optimized before the surgical incision. Surgical management consisted of craniotomy for debridement and elevation and additional hematoma evacuation; we had more than a half with an intraoperative finding of an underlying dural tear that got repaired (duroplasty), as similar to other studies,^27,30^ as most of the patients in our study were assault victims of potential by unknown robbers using a metallic bar with a high-energy rapid compression force applied to a small area of the skull.

### Surgical timing and outcomes

Overall, regarding the main outcomes of interest, the incidence of SSI was high; a total of 24/127 (18.9%) developed SSI within 3 months. This prevalence was high due to a relatively longer period of 3 months in comparison to other studies reporting mainly the occurrence of infection during the hospital main stay^27^; the most common type of SSI was superficial incisional infection. The two groups of interest (surgical intervention before versus after 48 h of injury) had different patterns of outcomes. The surgical intervention done after 48 h was associated with a higher rate of infection (*p* = 0.006), a longer median of postoperative hospital stay of 8 versus 5 days when compared with the surgical intervention done before 48 h (*p* < 0.001), and a higher rate of reoperation (*p* = 0.05). The rate of SSI infection was increasing as the number of days was added beyond 48 h, as shown in Figure 5. Another retrospective study found that there was no increased risk of SSI within 72 h of injury, even after primary bone replacement.^44^ Our result of the cutoff of a safe timing of surgery before 48 h to prevent SSI is supported by the retrospective study done by Jennett and Miller with compound DSFs to examine the causes and consequences of infection and to assess the effect of surgical timing (early vs. delayed)^42^; they concluded that the incidence of infection was significantly greater in patients with a 48-h delay between injury and surgery, as we found in our prospective study. This may answer the gap left by Bullock et al. in their compilation of the 10 selected retrospective series with the recommendation of early surgery to reduce the incidence of SSI and to clear state surgery done before 48 h.^6^ In the preoperative period, we postulate under reserve that surgical timing in TBI for more than 48 h from injury may contribute significantly to the SSI pathophysiology through a cytokine-mediated post-traumatic inflammatory response, the dysbiosis of the scalp skin microbiota, and other injury factors; the timing can be shortened by an early clinical decision.

### Bacteriological findings

In our study, most of the samples cultured in SSI were monoinfection, as they are in most surgical procedures and a few poly infections with increased morbidity.^45,46^ In a cross-sectional study done by Deng *et al*. previously in the same hospital in Uganda in 2017 among all neurosurgical cranial procedures in which TBI surgery represented about 61.2% of cases, monobacterial infection was the commonest bacteriological feature of SSI.^47^ This evidence may be linked to the climate setting of SSA and might not apply to countries with colder climates. We can postulate that African skin microbiota has predisposing factors to the occurrence of SSI, with the same being frequently cultured in the microbiology assays of SSI. Gram-negative microorganisms constituted the highest number of isolates, as found in that study by Deng *et al.*^47^ This resistance is also similar in most of the microbiologic studies of the biofilm bacterial communities, demonstrating resistance to routine antiseptic agents for surgical preparation.^46,48^ In our study, we used a wound/pus swab that is known to be less sensitive than real microbial biofilm studies. We had two samples of pus with no growth after 72 h, probably due to the effectiveness of the antibiotics, where dead bacteria colonies cannot grow in the disc. The central nervous system (CNS) mortality was 100% associated with polymicroorganism infections including *E. coli.*

### Predictors of the SSI

From the literature, with a vast majority of our old retrospective case series, it is noted that few factors contribute to the occurrence of SSI following the surgery of DSFs, mainly the wound status before surgery, the manipulation of bone fragments, associated dura tear, pneumocranium, and intracranial injuries in retrospective studies.^5,28,30^ The timing of surgery of more than 48 h has been known to be associated with increased risk in one of the retrospective series done about 4 decades ago.^42^ In our study, at bivariate analysis between the groups of early and delayed surgery, the delayed group was associated with a higher rate of infection (*p* = 0.006) probably due to the attributive factors of the presence of the air sinus involvement with the DSF (*p* = 0.025), a longer duration of anesthesia (*p* = 0.057), and a longer duration of the surgical procedure (*p* = 0.0194).

Considering only the group of SSI and no SSI at multivariate analysis, the parameters delayed surgical timing (95% CI: 1.25–6.22), the presence of pneumocranium (95% CI: 1.50–5.36) as found by Shao *et al*.,^5^ and involvement of air sinus (95% CI: 1.55–5.47) were associated with a more than a 2.5-fold increase in the rate of SSI. The postresuscitation GCS 9–13 (95% CI: 1.02–3.77) was associated with a 96% increase in the risk of SSI. Mukherjee et al. found in their prospective series with 49 surgically operated patients with compound DSF and followed up for 3 months for CNS infection among survivors at multivariate analysis adjusting for the confounders (age, sex, GCS, dural tear, and surgical intervention) that low GCS showed nonsignificant benefit with surgical intervention to prevent SSI.^28^ This study has a bias toward patient selection with minor injuries and severely sick patients. From our multivariate analysis, we summarized a simple-designed graphical model with size-weighted predictors of SSI in patients with DSF (Fig. 7). In this graphical model, the timing of surgery is the only modifiable parameter after the injury that can largely reduce the risk of the occurrence of SSI when it is decided within 48 h of the injury. This seems to reconcile with different previous studies advocating for early surgery of DSF without pressing the safety margin of the timing of surgery.^6,39,42,49^

### Effects of the surgical timing on the secondary outcomes

The increased postoperative duration was associated with delayed surgical timing (95% CI: 1.01–1.63), the presence of an underlying cerebral contusion (95% CI: 1.09–1.68), and the compound type of DSF (95% CI: 1.13–1.80). A major issue of availability of beds in high volume hospitals, in particular the length of hospital stay, was prolonged with delayed surgery (95% CI: 1.55–2.25), the presence of associated long bone fracture (95% CI: 1.03–1.61), and the compound type of DSF (95% CI: 1.14–1.64).

### Strength, clinical relevance, and generalizability of this study

The prospective design with a proper patient’s selection in this study in the SSA setting was very appropriate to inform the medical personnel about the risk outcomes of delayed surgery for patients with TBI with DSF in a follow-up period of 3 months and eventually its predictors. Most clinicians in SSA, especially in neurosurgery services, discharge their patients without a proper outpatient clinic follow-up plan. We found that our findings can be clinically relevant in the context of SSA, where the practice of neurosurgery is moving to a new era of reaching remote rural areas by referring patients to the main centers with a delay in obtaining brain imaging. We also state that our findings are generalizable, although we did not include some other perioperative variables in the analysis of this study such as the size of the DSF, the presence of drain, and other intraoperative details. Overall, we found the variables analyzed in the predictor modeling within this nearly homogenous cohort were consistent for analyzing the effect of the surgical timing on patients admitted with GCS above 8.

### Study limitations

Our study was done in a single center with a high volume of patients with TBI, and its results may reflect inerrant local practice as there is a big variation in the neurosurgical management of DSF based on the countries.^7^ We believe that our long-term experience with a high volume of patients with TBI with DSF and relatively standardized up-to-date neurosurgical practice may compensate for this fact, but a future collaborative multicenter study in several SSA countries using the same methodology with additional variables will confirm the generalizability of these findings. The allocation of surgical timing might have been biased with the neurosurgical decision based on the patients’ inherent characteristics, but our wide exclusion criteria in the study might have significantly counterbalanced these situations. Some injury parameters such as the real time of injury might not have been captured accurately, and we had to rely sometimes on the estimated time of the patient who last recalled being fully awake before the injury and reported the groups in brackets of days rather. The study happened just after the first wave of COVID-19 in Uganda; the delay in patients’ referral during the post-COVID-19 era in terms of mobility restriction, the readiness of the brain CT scan after some COVID-19 protocol of PCR testing, the readiness of operating theaters in busy settings of high volume trauma care, and also frequent fumigation of the operating theater after suspicion of COVID-19 contamination have contributed largely to the exaggeration in the delayed surgical timing with frequent theater list rescheduling for the so-called cold-cases during the COVID-19 emergency crisis in health systems.^50,51^ On the other side of this unfortunate health system situation, referral challenges during the pandemic have increased the odds of the prediction of SSI in prolonged delayed surgery.

## Conclusions

The surgical timing of DSF after 48 h of injury is significantly associated with a higher incidence of SSI and eventually a longer hospital length of stay. The preoperative predictors of SSI following surgery of DSF among hemodynamically stable patients with TBI with GCS above 8 were the surgical timing after 48 h of injury, the frontal location involving the opening through the air sinus, and the presence of pneumocranium on the head CT scan. The surgical timing is the only modifiable predictor after injury. We recommend surgical intervention before 48 h of injury to reduce the risk of SSI and hospital stay in SSA, mainly by obtaining timely brain CT and early referral of patients with TBI to the nearby neurosurgical center. We also advocate for a revision of the antibiotic protocol in SSA based on the patterns of microbial resistance to commonly prescribed antibiotics in cases when having nonmodifiable predictors of SSI for DSF surgery.

## Supplementary Material

Supplementary Table S1

Supplementary Table S2

## Data Availability

The datasets generated and analyzed during the current study are not publicly available due to legal and ethical reasons but are available from the corresponding author on reasonable request.

## Abbreviations Used

AIRR: Adjusted incidence rate ratio
ARR: Adjusted risk ratio
CI: Confidence interval
CT scan: Computerized tomography scan
DSF: Depressed skull fracture
GCS: Glasgow coma scale
IQR: Interquartile range
MNRH: Mulago National Referral Hospital
RTC: Road traffic Crashes
SSA: Sub-Saharan Africa
SSI: Surgical Site Infection
TBI: Traumatic brain injury
UIRR: Unadjusted incidence rate ratio
URR: Unadjusted risk ratio
