# Supplementary material for: Timing of Surgery and Preoperative Predictors of Surgical Site Infections for Patients with Depressed Skull Fractures in a Sub-Saharan Tertiary Hospital: A Prospective Cohort Study
Source: Neurotrauma Rep. 2024 Sep 20;5(1):824–44. doi: 10.1089/neur.2024.0088 (PMC11462418; doi:10.1089/neur.2024.0088)
Supplement: Supplementary Table S1 [file neur.2024.0088_Supplementary_TableS1.pdf]

Supplementary material 1: Sub-analytic distribution of the participants' demographics and clinical presentation by surgical timing within the group of surgical intervention of depressed skull fracture done in ≤ 48 hours.

**Variable**	**Surgical timing within ≤ 48 hours**		**Fisher's exact**
	**<24 hours**<br>**N (Col%); 45 (69.2%)**	**24-48 hours**<br>**N (Col%); 20 (30.8%)**	**p-value**
**Age** (Years), median (IQR); Mann-Whitney test	23 (14-29)	25.5 (21.5- 31.5)	0.1611
**Sex**			
Male	3 (50.0%)	3 (50.0%)	0.361
Female	42 (71.2%)	17 (28.8%)	
**Mechanism of injury**			
Assault	25 (69.4%)	11 (30.6%)	0.057
Pedestrian Knocked RTC	12 (92.3%)	1 (7.7%)	
passenger motorcycle RTC	6 (60.0%)	4 (40.0%)	
Others	2 (33.3%)	4 (66.7%)	
**Long bones fracture (Poly trauma)**			
No	43 (68.3%)	20 (31.7%)	1.000
Yes	2 (100%)	0	
**Type of DSF**			
Simple	23 (71.9%)	9 (28.1%)	0.789
Compound	22 (66.7%)	11 (33.3%)	
**Post-resuscitation admission GCS**			
9-13	12 (66.7%)	6 (33.3%)	0.773
14-15	33 (70.2%)	14 (29.8%)	
**Neurological focal deficit**			
No deficit	36 (70.6%)	15 (29.4%)	0.747
Deficit	9 (64.3%)	5 (35.7%)	
**Post-traumatic seizures**			
No	34 (69.4%)	15 (30.6%)	1.000
Yes	11 (68.8%)	5 (31.3%)	
**Pre-operative ASA classification**			
Class 1	3 (75.0%)	1 (25.0%	0.894
Class 2	33 (70.2%)	14 (29.8%)	
Class 3	9 (64.3%)	5 (35.7%)	
**Pre-operative hemoglobin**			
>12	28 (70.0%)	12 (30.0%)	0.926
10-11	11 (64.7%)	6 (35.3%)	
<10	6 (75.0%)	2 (25.0%)	

NOTE: *IQR – Inter Quartile Range*
