# Supplementary material for: Timing of Surgery and Preoperative Predictors of Surgical Site Infections for Patients with Depressed Skull Fractures in a Sub-Saharan Tertiary Hospital: A Prospective Cohort Study
Source: Neurotrauma Rep. 2024 Sep 20;5(1):824–44. doi: 10.1089/neur.2024.0088 (PMC11462418; doi:10.1089/neur.2024.0088)
Supplement: Supplementary Table S2 [file neur.2024.0088_Supplementary_TableS2.pdf]

Supplementary material 2: Sub-analytic distribution of the participants' head CT findings, management & outcomes by surgical timing within the group of surgical intervention of depressed skull fracture ≤ 48 hours.

**Variable**	**Surgical Timing within ≤ 48 hours**		**Fisher's exact**
	**<24 hours**<br>**N (Col%); 45 (69.2%)**	**24-48 hours**<br>**N (Col%); 20 (30.8%)**	**p-value**
**Location of DSF**			
Frontal	13 (56.5%)	10 (43.5%)	**0.040**
Frontal parietal	11 (78.6%)	3 (21.40%)	
Frontal temporal	4 (100.0%)	0	
Parietal	14 (87.5%)	2 (12.50%)	
Temporal	3 (37.50%)	5 (62.50%)	
**Underlying extra axial hemorrhage**			
No	29 (63.0%)	17 (37.0%)	0.140
Yes	16 (84.2%)	3 (15.8%)	
**Underlying cerebral contusion**			
No	9 (75.0%)	3 (25.0%)	0.741
Yes	36 (67.9%)	17 (32.1%)	
**Pneumocranium**			
No	41 (70.7%)	17 (29.3%)	0.667
Yes	4 (57.1%)	3 (42.9%)	
**Air sinus involvement**			
No	43 (71.7%)	17 (28.3%)	0.165
Yes	2 (40.0%)	3 (60.0%)	
**Basal cisterns in radiological finding**			
Absent	2 (100.0%)	0	0.617
Open	28 (71.8%)	11 (28.2%)	
Compressed	15 (62.5%)	9 (37.5%)	
**Midline shift > 5mm**			
No	41 (68.3%)	19 (31.7%)	1.000
Yes	4 (80.0%)	1 (20.0%	
**Intraop. finding of underlying dural tear**			
No	23 (69.7%)	10 (30.3%)	1.000
Yes	22 (68.8%)	10 (31.3%)	
**Duration of Anesthesia** (minutes), median (IQR); Mann-Whitney test	110 (95-130)	132.5 (95-172.5)	0.1879
**Duration of surgical procedure** (minutes), median (IQR); Mann-Whitney test	70 (60-90)	80 (55-107.5)	0.3468
**Surgical site infection (SSI)**			
No	40 (67.8%)	19 (32.2%)	0.657
Yes	5 (83.3%)	1 (16.7%)	
**Hospital outcomes after the initial surgery**			
Improved	40 (69.0%)	18 (31.0%)	0.833
Re-operated	2 (100%)	0	
Prolonged stay	3 (60.0%)	2 (40.0%)	
**Post Operation length of stay** (Days) median (IQR); Mann-Whitney test p-value	3 (3-5)	4 (3-4.5)	0.4454

NOTE: *IQR – Inter Quartile Range*
